# Supplementary material for: Unveiling the cell biology of hippocampal neurons with dendritic axon origin
Source: J Cell Biol. 2024 Nov 4;224(1):e202403141. doi: 10.1083/jcb.202403141 (PMC11536041; doi:10.1083/jcb.202403141)
Supplement: Table S1 — lists reagents and resources. [file JCB_202403141_TableS1.docx]

**Reagents and Resources**

| REAGENT or RESOURCE | SOURCE | IDENTIFIER |  |
| --- | --- | --- | --- |
| Antibodies | | |  |
| anti-AnkG  (mouse, dilution 1:500) | Neuromab | Cat#N106_36;  RRID:AB_2877524 |  |
| anti-βIV-Spectrin  (mouse, dilution 1:200) | Neuromab | Cat#75-377;  RRID:AB_2315818 |  |
| anti-βIV-Spectrin  (rabbit, dilution 1:300) | M.N. Rasband | Self-made |  |
| anti-neurofascin  (mouse, dilution 1:200) | Neuromab | Cat#75-172;  RRID:AB_2282826 |  |
| anti-TRIM46  (rabbit, dilution 1:500) | Synaptic Systems | Cat#377003  RRID:AB_2631232 |  |
| anti-tyrosinated α tubulin  (mouse, dilution 1:500) | Synaptic system | Cat# 302 117  RRID:AB_2620047 |  |
| anti-acetylated α tubulin  (mouse, dilution 1:500) | Santa Cruz | Cat#sc-23950  RRID:AB_628409 |  |
| anti-MAP2-Alexa Fluor-488  (mouse, dilution 1:500) | Merck Millipore | Cat#MAB3418X;  RRID:AB_11212966 |  |
| anti-MAP2  (chicken, dilution 1:400) | Synaptic Systems | Cat#188 006;  RRID: AB_2619881 |  |
| anti-synaptopodin  (rabbit, dilution 1:500) | Synaptic Systems | Cat#163002;  RRID:AB_887825 |  |
| anti-gephyrin  (mouse, dilution 1:500) | Synaptic Systems | Cat#147 011  RRID: AB_887717 |  |
| anti-gephyrin  (chicken, dilution 1:500) | Synaptic Systems | Cat#147 009  RRID: AB_2943527 |  |
| anti-homer-1  (mouse, dilution 1:500) | Synaptic Systems | Cat#160 011;  RRID:AB_2120992 |  |
| anti-VGAT  (mouse, dilution 1:500) | Synaptic Systems | Cat# 131 011;  RRID:AB_887872 |  |
| anti-VGLUT1  (guinea pig, dilution 1:500) | Synaptic Systems | Cat#135304;  RRID:AB_887878 |  |
| anti-pan-Nav1  (mouse, dilution 1:500) | Neuromab | Cat# 75-405;  RRID: AB_2491098 |  |
| anti-Brevican-Alexa 594  (mouse, dilution 1:200) | Neuromab | Cat# 75-281;  RRID:AB_2315822 |  |
| anti-mouse-Alexa Fluor 568  (dilution 1:500) | Thermo Fisher Scientific | Cat#A-11004;  RRID:AB_2534072 |  |
| anti-mouse-Alexa Fluor 647  (dilution 1:500) | Thermo Fisher Scientific | Cat#A-21235;  RRID:AB_2535804 |  |
| anti-rabbit-Alexa Fluor 568  (dilution 1:500) | Thermo Fisher Scientific | Cat#A-11036;  RRID:AB_10563566 |  |
| anti-mouse-Abberior Star 580  (dilution 1:500) | Abberior | Cat#2-0002-005-1;  RRID:AB_2620153 |  |
| anti-guinea pig-Abberior Star 635p  (dilution 1:500) | Abberior | Cat# ST635P-1006-500UG;  RRID:AB_2893230 |  |
| anti-mouse-Nanofluor2X-AbberiorStar635p (dilution 1:500) | Nanotag | Cat#N2002;  RRID: N/A |  |
| anti-mouse-Nanofluor2X-AbberiorStar580 (dilution 1:500) | Nanotag | Cat# N2702;  RRID: AB_2936181 |  |
| anti-mouse-Alexa594  (dilution 1:200) | Thermo Fisher Scientific | Cat# A-11032  RRID: AB_2534091 |  |
| anti-chicken-CY3 | Jackson ImmunoResearch Labs | Cat# 103-165-155  RRID: AB_2337386 |  |
| anti-chicken-Alexa488 | Thermo Fisher Scientific | Cat# A11039  RRID:AB_2534096 |  |
| anti-mouse-Alexa555 | Thermo Fisher Scientific | Cat# A31570  RRID: AB_2536180 |  |
| anti-rabbit-CF680 | Biotium | Cat# 20067-1  RRID: AB_10871686 |  |
| Recombinant DNA constructs | | | |
| CMV-EB3-TdTomato | Addgene | Cat#50708  RRID: Addgene_50708 |  |
| EGFP-Rab3A | Addgene | Cat#49542  RRID: Addgene_49542 |  |
| NPY-mEGFP |  | Schlager et al, 2010  DOI: 10.1038/emboj.  2010.51 |  |
| pAAV-syn-EGFP-Rab3A | Yuhao Han | this study |  |
| pAAV-syn-LAMP1-mCherry |  | van Bommel et al, 2019  DOI: 10.15252/embj.  2018101183 |  |
| Bacterial and Virus Strains | | | |
| *E. coli* XL10Gold | Agilent | Cat#200314 |  |
| rAAV9-syn-EGFP-Rab3A | UKE vector facility | this study |  |
| rAAV9-syn-LAMP1-mCherry | UKE vector facility | van Bommel et al, 2019 |  |
| Experimental Models: Organisms/Strains | | | |
| Rat: Wistar Unilever | Envigo/UKE animal  facility | HsdCpb:WU |  |
| Primers & Enzymes | | | |
| rAAV9-Syn-EGFP-Rab3A Forward (5’-3’):  GACTCAGATCTCGAGCTCAAGCTTTAG  GAATGGCATCCGCCACAGACTC | IDT | this study |  |
| rAAV9-Syn-EGFP-Rab3A Reverse (5’-3’):  GTTGATTATCGATAAGCTCATATGTCAG  CAGGCGCAGTCCTGGT | IDT | this study |  |
| HindIII | Thermo Fisher Scientific | Cat#ER0501 |  |
| FastDigest NdeI | Thermo Fisher Scientific | Cat#FD0583 |  |
| Hibrid DNA Polymerase | Roboklon | Cat#E2950-01 |  |
| Critical Commercial Assays |  |  |  |
| CF®640R Mix-n-Stain™ antibody  labelling kit | Biotium | Cat#92245 |  |
| Cold Fusion Cloning Kit | BioCat | Cat#MC010A-NCC-SBI |  |
| Chemicals and ligands | | | |
| Phalloidin-atto647N | Sigma-Aldrich | Cat#65906-10NMOL |  |
| Phalloidin-Alexa647+ | Thermo Fisher Scientific | Cat#A30107 |  |
| Lipofectamine 2000 | Invitrogen | Cat#11668027 |  |
| Transferrin-568 | Thermo Fisher Scientific | Cat#T23365 |  |
| Tetrodotoxin | Roth | Cat#6973.1 |  |
| Sodium Chloride | VWR Chemicals | Cat#27810.364 |  |
| Potassium Chloride | Roth | Cat#6781.1 |  |
| ROTI Histofix | Roth | Cat#A146.6 |  |
| Trypsin (0.25%) | Thermo Fisher Scientific | Cat#25200-056 |  |
| Poly-l-lysine | Sigma-Aldrich | Cat#P2636 |  |
| Glutamine | Thermo Fisher Scientific | Cat#25030024 |  |
| EM grade PFA (32%) | Electron Microscopy  Science | Cat#15714 |  |
| Smart Kit | Abbelight |  |  |
| 2-mercaptoethanol | Sigma | Cat#30070 |  |
| Medium and supplements | | | |
| BrainPhys+SM1 Supplement | StemCell | Cat#05790 |  |
| Neurobasal+B27 | Thermo Fisher Scientific | Cat#21103049) |  |
| B27 | Thermo Fisher Scientific | Cat#17504044 |  |
| DMEM | Sigma | Cat#D6429-500ML |  |
| FBS | Thermo Fisher Scientific | Cat#A4766801 |  |
| Pen/Strep | Thermo Fisher Scientific | Cat#15070063 |  |
| Software and Algorithms | | | |
| (Fiji is just) ImageJ Versions 1.49v & 1.53t |  | <http://fiji.sc/>;  RRID:SCR_002285 |  |
| Prism v7.03 | GraphPad | <https://www.graphpad.com/scientific-software/prism/> |  |
| Spyder 4.2.5 | Pierre Raybaut & Carlos Cordoba | https://www.spyder-ide.org/ |  |
| Python 3.8.8 | Python | https://www.python.org/about/ |  |
| R 4.3.0 | R | https://www.r-project.org/ |  |
| Peak Cal 3.0 | Yuhao Han | https://github.com/HU-Berlin-Optobiology/AIS-project.git |  |
| AIS Pack 4.0 | Yuhao Han | https://github.com/HU-Berlin-Optobiology/AIS-project.git |  |
| KYMOA 6.0 | Yuhao Han | https://github.com/HU-Berlin-Optobiology/AIS-project.git |  |
| KA Post Processing 2.0 | Yuhao Han | https://github.com/HU-Berlin-Optobiology/AIS-project.git |  |
| Synpo_det_1.0 | Yuhao Han | https://github.com/HU-Berlin-Optobiology/AIS-project.git |  |
| AIS_synpo_cluster_analysis | Yuhao Han | https://github.com/HU-Berlin-Optobiology/AIS-project.git |  |
| Intensity_measurement_batch | Yuhao Han | https://github.com/HU-Berlin-Optobiology/Optobio |  |
| ScanSlide_stitching_marco_3.3 | Erich Weisheim | https://github.com/HU-Berlin-Optobiology/Optobio |  |
